# Comorbidities and Vaccination Status of COVID-19 All-Cause Mortality at a Tertiary Care Center of Western India

**DOI:** 10.7759/cureus.21721

**Published:** 2022-01-30

**Authors:** Manoj Verma, Savitri Sharma, Arun Kumar, Afzal Hakim, Suman Bhansali, Rita Meena

**Affiliations:** 1 Community Medicine, Dr. Sampurnanand (S. N. Medical College), Jodhpur, IND

**Keywords:** breakthrough infection, covaxin, covishield, covid, corona

## Abstract

Introduction: COVID-19 vaccines have been found to be efficacious for preventing severe disease, yet breakthrough infections and deaths have occurred in a small proportion of vaccinated individuals. This study aimed to describe the vaccination status and comorbidities of COVID-19 all-cause deaths.

Methods: This descriptive observational study was conducted at a tertiary care center in western India. A total of 310, RT-PCR positive COVID-19 deaths, aged 45 years and above irrespective of the cause of death (all-cause mortality), were included in the study. Death after breakthrough infection was defined as death in patient with disease onset after 14 days of the second dose of vaccine.

Results: Diabetes was the most common comorbidity found in 17.1% of the deaths, followed by hypertension. Cardiovascular disease and renal disease were other common comorbidities seen in 8.7% and 4.83% deaths respectively. Other less common comorbidities include neurological disorders, HIV, autoimmune disorders. Out of these 310 deaths, 21.4% of patients developed disease within 14 days of the first dose. Death after true breakthrough infection (after 14 days of both doses) was seen in only two patients (0.6%). One of these two patients was aged 60 years and had diabetes, while the other was aged 72 years and had a history of smoking.

Conclusion: Diabetes and hypertension were the most common comorbidities, indicating a higher risk of mortality among comorbid patients. Only a small proportion of deaths (0.6%) occurred after breakthrough infection beyond 14 days of two doses. COVID-19 vaccines have shown promising efficacy against severe disease, thus high vaccination coverage needs to be achieved to prevent morbidity and mortality.

## Introduction

COVID-19 pandemic, caused by the novel SARS-CoV-2 virus, started in December 2019 and has been responsible for high morbidity and mortality globally [1]. Many countries witnessed their second wave after an intermediary decline, including India [2]. More than 25 million cases had been reported by India, with a peak of more than 400,000 cases on a single day on May 7, 2021 [3].

Numerous control measures like social distancing, use of face masks, and nationwide lockdowns have been effective to varying extents in slowing down transmission, but COVID-19 vaccination is expected to be the most effective and fastest way to stop the pandemic [4]. COVID-19 vaccines have been developed in record time and are being seen as a critical tool in managing the ongoing COVID-19 pandemic [1,5,6].

Numerous trials have found vaccines to be safe, immunogenic, and efficacious in preventing symptomatic and severe diseases [5,7]. Studies from India have also proven the efficacy of Covishield and Covaxin vaccines [3,8]. A single dose of Covishield had an efficacy of 76.0% (95% CI 59.3 to 85.9) against symptomatic COVID-19 in the first 90 days after vaccination [6], while after the second dose vaccine efficacy reaches up to 81.3% [6], Vaccination with these two vaccines in India has been started from January 16, 2021 after the Emergency Use Approval [3].

Mortality due to COVID-19 has been reported to be relatively high in older patients [9], especially those with comorbidities like diabetes, hypertension, cardiovascular disease, cancer, etc. [10-13], thus vaccination in 60 years and above population was started early followed by those age above 45 years. Despite the high level of vaccine efficacy, a small percentage of fully vaccinated persons will develop infections and symptomatic COVID-19 [5], and in a minority, death can also occur. Also, the emergence of various new strains has been shown to escape immune mechanism [14] and can lead to vaccine failure.

Epidemiology of COVID-19 cases and deaths are expected to vary during the second wave because of the initiation of vaccination, the spread of emerging SARS-CoV-2 variants, and overburdened healthcare systems [15]. Hence, the present study was conducted with the objective to describe the comorbidities and vaccination status of COVID-19 positive all-cause mortalities at a tertiary care center in western India.

## Materials and methods

This descriptive observational study was conducted at the Department of Community Medicine, at a Medical College associated tertiary care center in western India. This center is the largest multi-specialty hospital catering a population over 35 lakh population of the district and also acts as the referral center for neighboring districts. During the study period, nearly 200,000 samples were tested at the center, of which over 50,000 samples were found positive for COVID-19. Among these, 4,654 patients were admitted at this center. From these admitted cases, RT-PCR positive COVID-19 deaths irrespective of the cause of death (all-cause mortality), were included in the study. COVID-19 positive deaths linked to incidental causes like road traffic accidents with severe injury, suicide, or poisoning were excluded. Electronic records of all COVID-19 positive patients, residents of the catered district, aged 45 years and above, of either gender, who died while being admitted to the hospital, from March 15, 2021 to May 30, 2021 were included in the study. This period corresponds to the major part of the second wave of COVID-19 and 15 days after initiation of vaccination for the above 45 years population.

The study was started after approval from the Institutional Ethics committee. Data were accessed from the control room established for COVID-19 reporting at the Medical College level. Details were collected regarding general demographic characteristics (age, gender, residence), comorbidities (diabetes, hypertension, or any other systemic illness). Missing data were obtained from case sheets of patients accessed from the medical record room. Vaccination details were obtained by contacting the relatives telephonically. Verbal consent was obtained from these relatives before inquiring about any details. Complete information including vaccination details could be ascertained from a total of 310 subjects, which were finally included in the analysis. Death after breakthrough infection was defined as death in patients with disease onset after 14 days of the second dose of vaccine.

Statistical analysis

Categorical variables were summarized as frequency and percentage and were represented using appropriate graphs. The Chi-square test was used for the analysis of categorical variables. Statistical analysis was done using Epi info version 7.2.1.0 statistical software.

## Results

A total of 310 COVID-19 positive deaths were included in the study. There were more males (55.5%), with a male:female ratio of 1.24, with no difference in the age distribution (Table 1). 

**Table 1:** Age and gender distribution of study participants

Age (years)	Female		Male		Total		P-value
	N	%	N	%	N	%	
45-59	48	34.8	74	43.0	122	39.4	
60-74	64	46.4	73	42.4	137	44.2	0.660
75-89	24	17.4	23	13.4	47	15.2	
>90	2	1.4	2	1.2	4	1.3	
Total	138	100.0	172	100.0	310	100.0	

Diabetes was the most common comorbidity found in 17.1% of deaths, followed by hypertension (9.68%). Cardiovascular disease and renal disease were other common comorbidities seen in 8.71% and 4.83% deaths respectively. Other less common comorbidities include neurological disorders, HIV, autoimmune disorders. No significant difference was seen in the frequency of comorbidities among male and female patients except renal disease (Table 2).

**Table 2:** Frequency of various comorbidities and their relation to gender COPD - Chronic obstructive pulmonary disease, TB - tuberculosis

	Total (N=310)	Female (N=138)	Male (N=172)	P-value
Diabetes	53 (17.1%)	28 (20.3%)	25 (14.5%)	0.236
Hypertension	30 (9.68%)	16 (11.6%)	14 (8.1%)	0.407
Cardiovascular	27 (8.71%)	9 (6.5%)	18 (10.5%)	0.307
Renal	15 (4.83%)	2 (1.4%)	13 (7.6%)	0.012
Asthma/COPD	6 (1.94%)	4 (2.9%)	2 (1.2%)	0.492
TB	2 (0.65%)	1 (0.7%)	1 (0.6%)	0.577
Obesity	7 (2.26%)	6 (4.3%)	1 (0.6%)	0.067
Thyroid disorder	2 (0.65%)	1 (0.7%)	1 (0.6%)	0.577
Malignancy	2 (0.65%)	1 (0.7%)	1 (0.6%)	0.577
Others	15 (4.83%)	7 (5.1%)	8 (4.7%)	0.848

**Figure 1:**
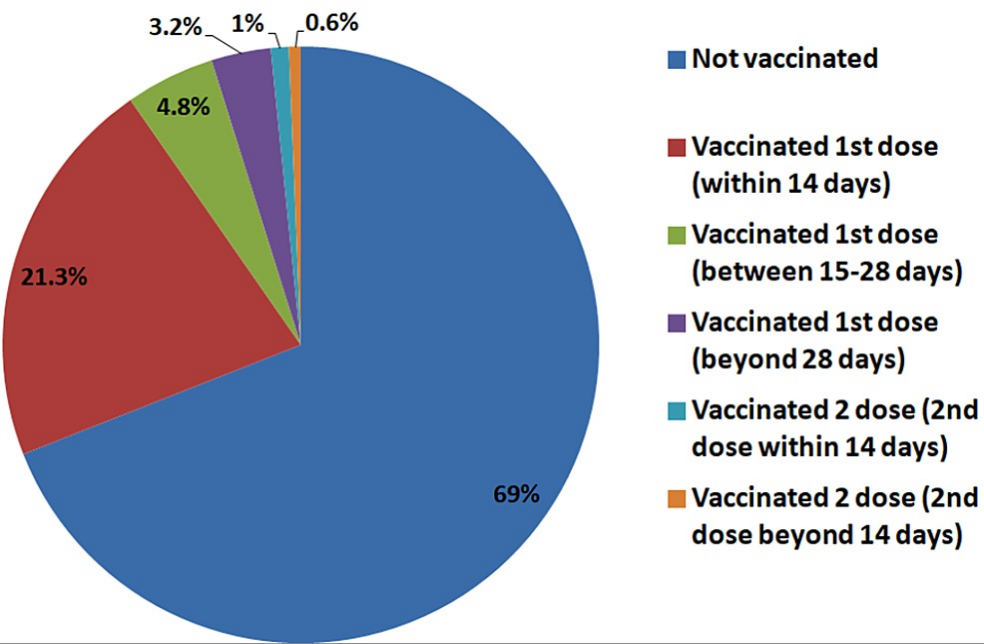
Vaccination status of COVID-19 deaths

Out of these 310 deaths, 214 (69%) were unvaccinated, while 66 (21.3%) patients developed the disease within 14 days of the first dose (21.4%). True breakthrough infection (after 14 days of the second dose) was seen in only two patients (0.6%) (Figure 1).

One of these two patients was aged 60 years and had diabetes, while the other was aged 72 years without any comorbidity but with a long history of smoking. The type of vaccine taken could be ascertained only from 81 out of 96 cases who had received at least one dose of vaccine, 74 had received Covishield, and seven had received Covaxin.

## Discussion

The present study involved COVID-19 deaths (all-cause mortality) aged 45 years or more to retrospectively look at its relation with COVID-19 vaccination status, which gives indirect evidence of the usefulness of mass vaccination in the actual population. The male:female ratio in the present study was 1.24:1. Other studies have also reported a higher proportion of deaths among males [16]. Low mortality among females could be due to X chromosome and sex hormones, which play a vital role in providing innate and adaptive immunity, while behavioral factors like smoking and alcohol could contribute to higher mortality among males [16].

A higher risk of death among older patients could be due to decreased immune response due to immunosenescence and more comorbidities [12]. The presence of any comorbidities has been found to be as high as 85% among COVID-19 deaths in past studies [13,16].

In the present study, diabetes (17.1%) and hypertension (9.35%) were the most common comorbidities among COVID-19 positive deaths. Past studies have reported arterial hypertension was the most prevalent chronic condition among deaths ranging from 17% to 65%. while diabetes has been found in 15.4% to 62% of deaths in studies [11,13,16-18].

In the present study, cardiovascular disease was seen in 8.7% deaths, while renal disease as a comorbidity was found in 4.7% of deaths. Similar studies have reported the coexistence of cardiovascular disease in 12.4% to 34.6% of deaths [13,16,17], while chronic renal failure was reported among 11.5% of decedents, in a recent study [17].

Obesity was found in 2.6% of deaths in the present study. Similar studies have reported obesity in up to 38.5% of deaths [17]. Raised BMI has been established as a risk factor for COVID-19 related mortality [18]. Chronic obstructive pulmonary disease (COPD) and other lung diseases have been reported as comorbidities in various studies, ranging from 10.6% to 23.1% of deaths [11,13,17]. Malignancy was found in nearly 1% of deaths in the present study. Malignancy as comorbidity had been reported in 1.5% to 10.2% deaths [11,13].

These comorbidities are among the most common independent causes of death globally. People with these morbidities usually have a poor immune system, which increases their susceptibility to infection, severe disease, and even death [13]. The reason for the relatively low prevalence of certain comorbidities in this study could be due to early preferential vaccination of those with comorbidities leading to less mortality among these patients and/or higher severity of disease even among persons without comorbidity probably due to the virulence of the variant of SARS-CoV-2 causing the second wave.

In the present study, two patients died after developing disease beyond 14 days of two doses (breakthrough infection), while another 13 (4.2%) developed disease 14 days after a single dose of vaccine.

Covishield vaccine has been shown to have high efficacy against severe disease [6]. In a multicentric phase-III trial, single-dose Ad26.COV2.S vaccine reported efficacy ranging from 66.9% to 85.4% against moderate to severe-critical disease with onset ≥14 days or ≥28 days after vaccine [1]. Vaccine efficacy was found to be lower among participants 60 years of age or older with coexisting conditions with onset at least 28 days after administration [1]. The multicentric trial study had reported three deaths in the vaccinated group, though none was reported to be COVID-19 related [1].

No vaccine is 100% effective and ‘Breakthrough cases’ are expected, especially before population immunity reaches sufficient levels to further decrease transmission [5], and a small proportion of these are expected to die. In the Pan-India cross-sectional COVAT study, Breakthrough infections were noted in 5.5% of Covishield and 2.2% of Covaxin recipients [3].

In the USA, more than 10,000 SARS-CoV-2 vaccine breakthrough infections have been reported with, a median age of 58 years [5]. More than 3000 patients have been reported to be hospitalized after breakthrough infection, of which 535 patients died [19]. Even these figures have been described to be an undercount of all SARS-CoV-2 infections among fully vaccinated persons as their National surveillance relies on passive and voluntary reporting, and data might not be complete or representative [19]. Oregon’s health authority has also reported more than 1,000 cases of breakthrough infections after vaccination; and death in 2% of these cases. Many of these cases were found to be due to different variants of concern [20].

The vaccine offers limited protection before 14 days of vaccination as indicated by the findings of the trial [1]. Cases occur within 14 days, and deaths among these cases can be expected to be higher when the vaccination campaign was started during the rising curve of the outbreak because many of the vaccinated individuals could already be infected and be in an incubation period and many individuals would get infection within next few days.

Also, coexisting conditions like diabetes, obesity, long-standing hypertension, and old age have been shown to reduce seropositivity after vaccination with either Covishield or Covaxin, indicating higher failure rates among these subjects [3].

Other studies have also shown decreased efficacy of vaccines against certain variants of SARS-CoV-2 [21-23]. Breakthrough infections after 14 days of two doses of mRNA vaccine have been reported due to SARS-CoV-2 variants of clinical concern [24]. From available genetic sequence data, the majority of the breakthrough infections in the United States were identified to be due to SARS-CoV-2 variants of concern [5]. The variant of concern, B.1.617.2 has emerged as one of the predominant variants in India and has shown increased transmissibility and immune escape [15,25]. A study from India also reported variant of concern B.1.617.2 as the predominant lineage among the 63 breakthrough cases that occurred after both Covishield and Covaxin [25]. An earlier study from Kerela found B.1.1.7 among most breakthrough cases and this strain has been shown to lower neutralizing antibody titers against Covishield [23].

The two deaths after breakthrough infection found in the present study could be simply due to primary vaccine failure (as no vaccine is 100% effective) or decreased efficacy among the elderly and those with comorbidities or infection with a variant of concern.

Population-based studies apart from the trials are needed to determine the true magnitude of vaccine efficacy, especially in relation to different phases of the pandemic and emerging strains. There is to identify the possible agent factors (e.g. mutations) [23] and human factors (e.g. age, comorbidities) responsible for breakthrough infections [4], especially severe disease and deaths, so find out possible remedies like change in the gap between two-dose or requirement booster with same or other types of vaccine or change in vaccine composition [26] with time and geography as is the case with influenza.

This study has some limitations. Information could be ascertained from only 310 deaths out of 700 COVID-19 positive all-cause mortality during the study period. Bias could be introduced as information about vaccination was obtained telephonically from relatives of deceased patients. Information regarding comorbidity for many patients was also obtained from relatives and could be underestimated.

## Conclusions

The findings of the present study give indirect evidence that death after breakthrough infection beyond two weeks of two doses of vaccine is rare. COVID-19 vaccines have shown promising efficacy, especially against severe disease, thus high vaccination coverage needs to be achieved to prevent morbidity and mortality and even possibly prevent any new “wave” of COVID-19. Diabetes and hypertension were the most common comorbidities among others and could indicate a higher risk of mortality among comorbid patients. Early and continuous research with a focus on emerging variants and patient characteristics like comorbidity are required to optimize preventive interventions like vaccine composition and schedules customized for those at higher risk.
